# Supplementary material for: Empirical methods for controlling false positives and estimating confidence in ChIP-Seq peaks
Source: BMC Bioinformatics. 2008 Dec 5;9:523. doi: 10.1186/1471-2105-9-523 (PMC2628906; doi:10.1186/1471-2105-9-523)
Supplement: Additional File 1 — A variety of html documents from the USeq web site detailing the available applications, their best usage, output file type descriptions, command line menus, etc. [file 1471-2105-9-523-S1.zip › USeqUserGuides/JavaDoc.zip!/JavaDoc/util/bio/annotation/Transcript.html]

Transcript


|  |  |  |
| --- | --- | --- |
| **Overview** \| **Package** \| **Class** \| **Use** \| **Tree** \| **Deprecated** \| **Index** \| **Help** | |  |
| **PREV CLASS**   **NEXT CLASS** | **FRAMES**    **NO FRAMES**     **All Classes** |  |
| SUMMARY: NESTED \| FIELD \| CONSTR \| METHOD | DETAIL: FIELD \| CONSTR \| METHOD |  |


---


## util.bio.annotation Class Transcript

```
java.lang.Object
  util.bio.annotation.Transcript
```

**All Implemented Interfaces:**: java.io.Serializable

---

``` public class Transcript extends java.lang.Object implements java.io.Serializable ```

Transcript relevant information, mRNA.

**See Also:**: Serialized Form

---

**Constructor Summary**

`Transcript(Gff3Feature f)`

`Transcript(int start,
int end,
java.lang.String name,
int orientation)`


| **Method Summary** | |
| --- | --- |
| `int` | `getEnd()` |
| `java.lang.String` | `getName()` |
| `int` | `getOrientation()` |
| `int` | `getStart()` |
| `java.lang.String` | `toString()` |

**Methods inherited from class java.lang.Object**

`equals, getClass, hashCode, notify, notifyAll, wait, wait, wait`

**Constructor Detail**

### Transcript

```
public Transcript(int start,
                  int end,
                  java.lang.String name,
                  int orientation)
```

---


### Transcript

```
public Transcript(Gff3Feature f)
```


**Method Detail**

### toString

```
public java.lang.String toString()
```

:   **Overrides:**: `toString` in class `java.lang.Object`

---


### getName

```
public java.lang.String getName()
```

---


### getOrientation

```
public int getOrientation()
```

---


### getEnd

```
public int getEnd()
```

---


### getStart

```
public int getStart()
```


---


|  |  |  |
| --- | --- | --- |
| **Overview** \| **Package** \| **Class** \| **Use** \| **Tree** \| **Deprecated** \| **Index** \| **Help** | |  |
| **PREV CLASS**   **NEXT CLASS** | **FRAMES**    **NO FRAMES**     **All Classes** |  |
| SUMMARY: NESTED \| FIELD \| CONSTR \| METHOD | DETAIL: FIELD \| CONSTR \| METHOD |  |


---
